# Supplementary material for: Canonical WNT signaling-dependent gating of MYC requires a noncanonical CTCF function at a distal binding site
Source: Nat Commun. 2022 Jan 11;13:204. doi: 10.1038/s41467-021-27868-3 (PMC8752836; doi:10.1038/s41467-021-27868-3)
Supplement: Supplementary file 1 — Supplementary Information [file 41467_2021_27868_MOESM1_ESM.pdf]

Supplementary Figures and Table

Canonical WNT signaling-dependent gating of *MYC* requires a non-canonical CTCF function at a distal binding site

Ilyas Chachoua, Ilias Tzelepis, Hao Dai, Jia-Pei Lim, Anna Lewandowska-Ronnegren, Felipe Beccaria Casagrande, Shuangyang Wu, Johanna Vestlund, Carolina Diettrich Mallet de Lima, Deeksha Bhartiya, Barbara Scholz, Mirco Martino, Rashid Mehmood and Anita Göndör

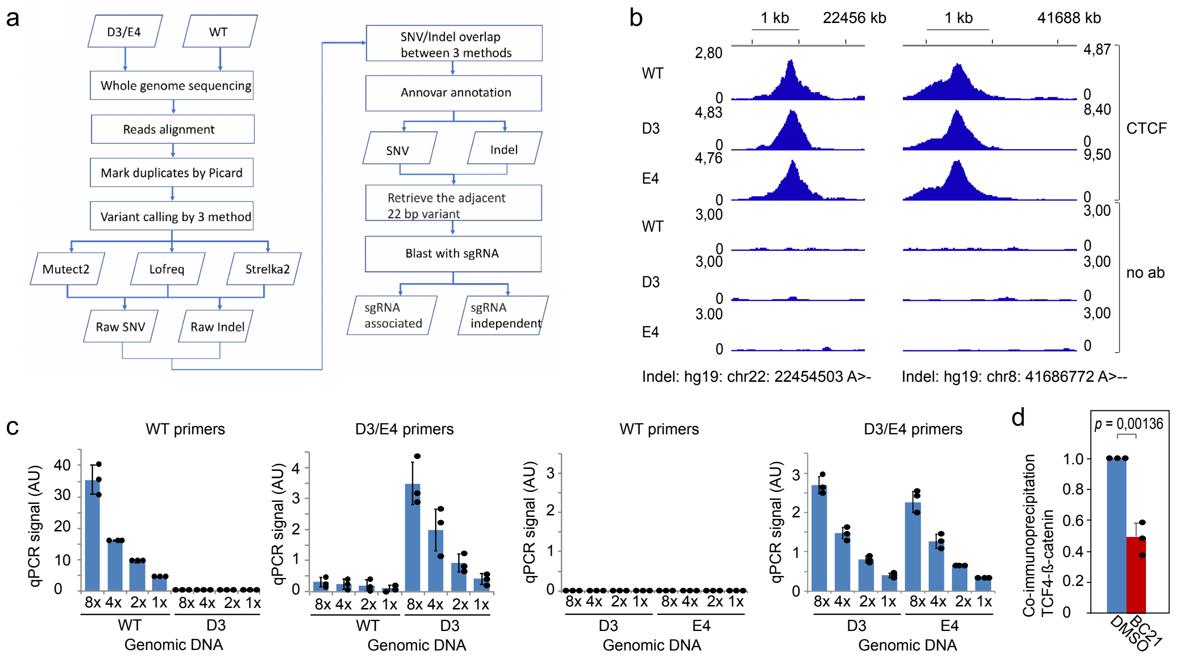

Supplementary Figure 1. Characterization of the D3 and E4 cell clones. a) Off-target detection pipeline modified from the GOTI method[22]. b) The distribution of two indels common to both D3 and E4 cells and close to CTCFBSs overlayered in ChIP-seq data (average of three independent samples). In both cases, the indels are positioned in the flank of the CTCFBSs. c) Titration of the allele-specific qPCR analyses to quantitatively discriminate between the WT and the D3 alleles using primers specific for either the WT or the mutant CTCFBSs (D3 and E4), respectively. d) Co immunoprecipitation analyses

of physical interactions between TCF4 and ß-catenin in the presence or absence of BC21. The data are normalized to the DMSO control. The bars in panels c and d represent the average of three independent samples with standard deviation. The *p* values were calculated by the two tailed Student's t-test.

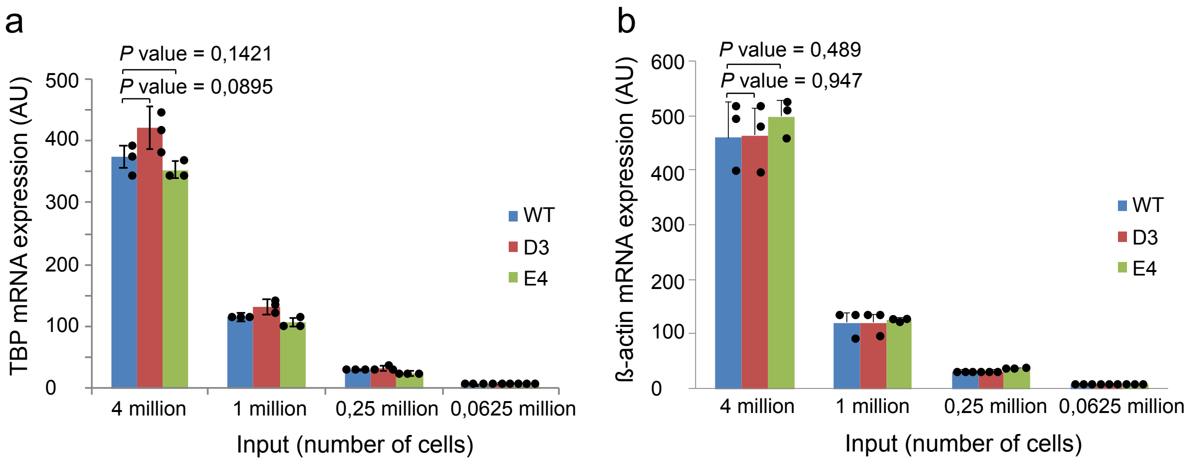

Supplementary Figure 2. Comparisons of TBP (a) and ß-actin (b) mRNA expression in relation to the input number of cells. The bars in panels a and b represent the average of three independent samples with standard deviation. The *p* values were calculated by the two tailed Student's t-test.

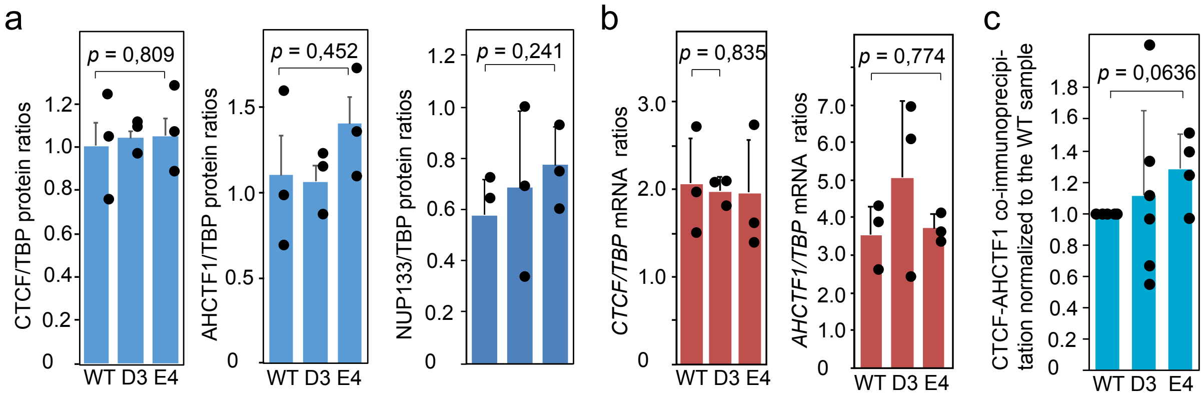

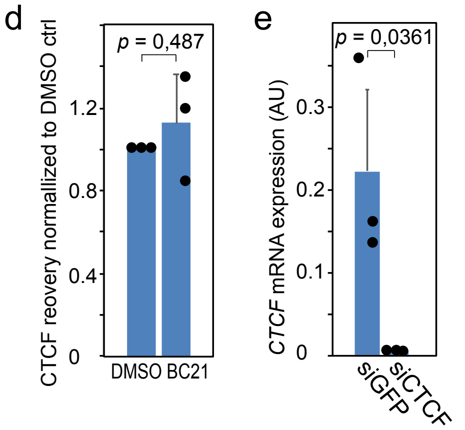

f

Group	Antibodies	Expt	Image	isPLA dots	Average distance (µm)	Arythmetic average (µm)	Weighted average (µm)
NT	AHCTF1/CTCF	1	1	2864	1,2053	1,46	1,54
			2	5888	1,7072		
		2	1	1908	1,2394	1,34	1,35
			2	2253	1,4465		
		3	1	4454	2,0131	1,93	1,93
			2	5514	1,9404		
			3	3858	1,8356		
DMSO	AHCTF1/CTCF	1	1	30682	1,7148	1,80	1,75
			2	6882	1,8909		
		2	1	4316	1,0970	1,25	1,31
			2	10358	1,3966		
		3	1	4625	1,9435	1,93	1,93
			2	3213	1,8994		
			3	4740	1,9349		
BC21	AHCTF1/CTCF	1	1	689	1,5048	1,63	1,75
			2	30	1,4855		
			3	1185	1,8924		
		2	1	807	1,2952	1,34	1,24
			2	431	1,7030		
			3	1179	1,0234		
		3	1	598	1,7685	1,90	1,88
			2	1442	1,8519		
			3	299	1,9268		
			4	576	2,0497		
NT	CTCF/NUP133	1	1	276	1,7000	1,37	1,33
			2	308	1,2025		
			3	594	1,2689		
			4	786	1,3003		

Supplementary Figure 3. Comparisons of the levels of AHCTF1 and CTCF expression/interactions in WT HCT-116, D3 and E4 cells. The relative expression levels of CTCF, AHCTF1 and NUP133 protein (a) or CTCF and AHCTF1 mRNA (b) in WT HCT-116, D3 and E4 cells were normalized to TBP expression. c) Relative difference in the recovery of CTCF-AHCTF1 co-immunoprecipitations in D3 and E4 cells normalized to the recovery of WT HCT-116 data. d) ChIP analyses of CTCF occupancy at the *CCAT1*-specific CTCFBS in control (DMSO) and BC21-treated WT HCT-116 cells. e) Efficiency of siRNA knock-down of CTCF mRNA expression. f) The average distribution of the CTCF-AHCTF1 and CTCF-NUP133 ISPLA signals in relation to the nuclear periphery. The bars in panels a-e represent the average of at least three independent samples with standard deviation. The *p* values were calculated by the two tailed Student's t-test.

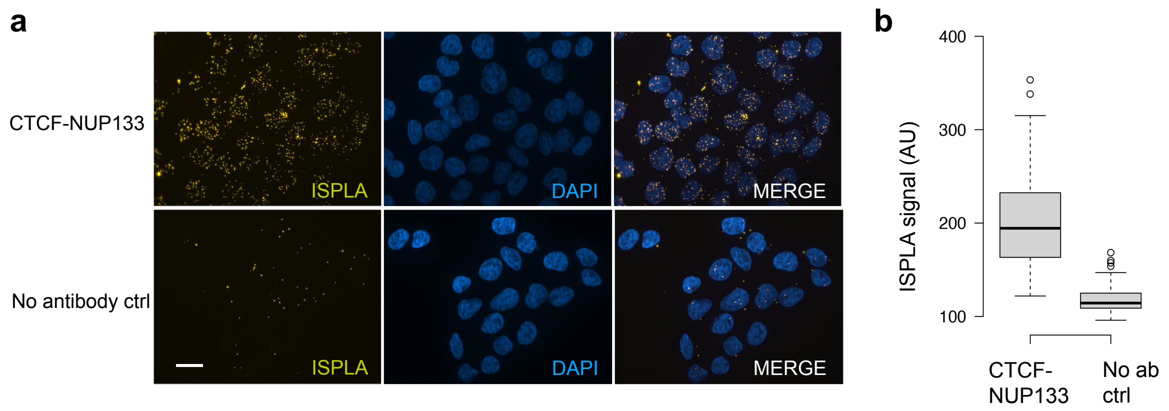

Supplementary Figure 4. *In situ* proximity analyses between CTCF and NUP133 in WT HCT-116 cells. a) Extended view images of the signals generated by the NUP133-CTCF ISPLA reaction. No ab ctrl = ISPLA performed in the absence of primary antibodies. Bar = 10 micrometer. b) The quantitation of the ISPLA signals representing the sum of two independent experiments was done as has been described previously[7]. Box-and-whisker plots show median values, interquartile ranges and Tukey whiskers.

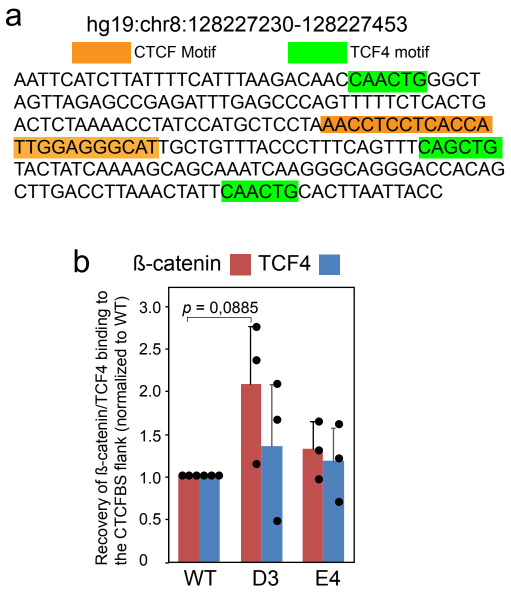

Supplementary Figure 5. ß-catenin and TCF4 binding to the *CCAT1*-specific CTCFBS region. a) The distribution of TCF4 binding motifs (marked in green) in the regions flanking the CCAT1-specific CTCFBS (marked in orange). b) ChIP analyses of ß-catenin and TCF4 binding to the *CCAT1*-specific CTCFBS region in WT HCT-116, D3 and E4 cells. The bars show in all instances the average of three independent experiments with indicated standard deviation. The *p* values were calculated by the two tailed Student’s t-test.

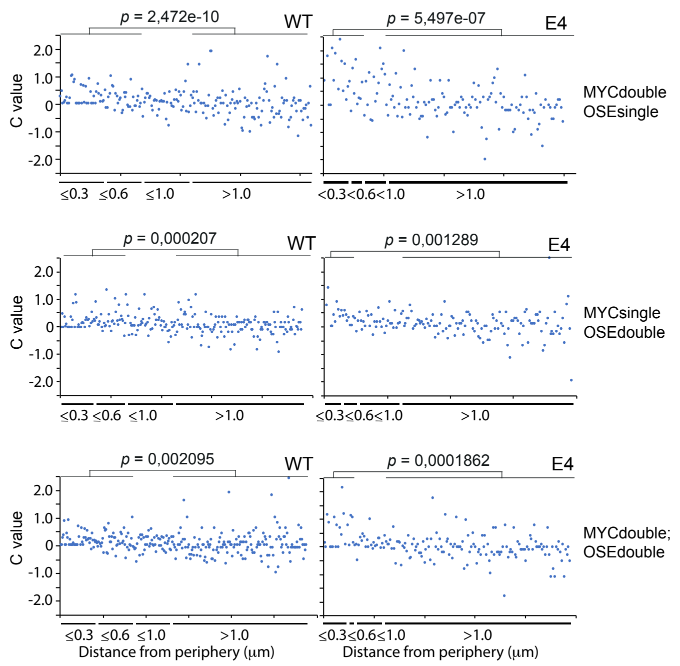

Supplementary Figure 6. The OSE-specific CTCFBS influences the proximity between the OSE, *MYC* and the nuclear periphery. Analysis of the "c" value (scoring for the difference in the proximity of the OSE and *MYC* to the nuclear periphery) in relation to the proximity between the OSE and the nuclear periphery in control and mutant HCT-116 cells for replicated alleles (MYCdouble/OSEsingle; MYCsingle/OSEdouble; MYCdouble/OSEdouble) (see **Fig. 5a and b** for additional information). A total of 1085 (Ctrl) and 740 (E4) alleles were counted from two independent experiments (*P* values: Two-sided KS test).

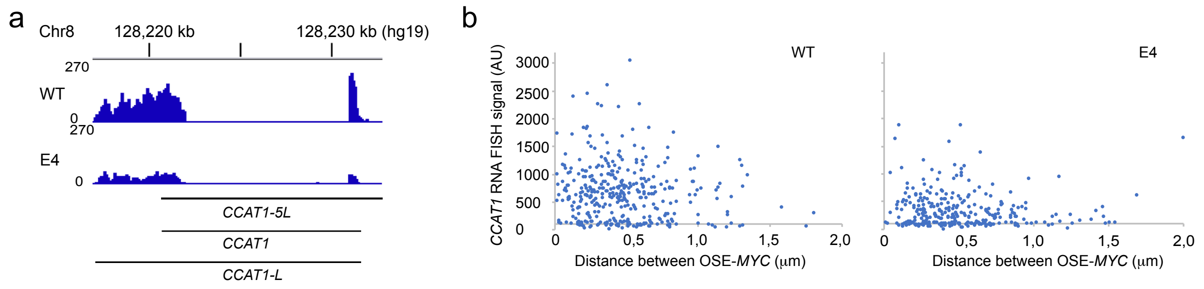

Supplementary Figure 7. *CCAT1* eRNA and *MYC* trafficking. a) Map (hg19) of *CCAT1* eRNA expression in WT and mutant (E4) HCT-116 cells. The y axes indicate the number of normalized reads. The previously reported[14,15] *CCAT1* eRNA variants are indicated. b) 3D DNA FISH analyses of the proximity between the OSE and *MYC* plotted against *CCAT1* eRNA FISH signals in WT HCT-116 cells and the E4 clone.

Supplementary Table I

qPCR primers for ChIP

**Locus**	Forward	Reverse	Cycle
MYC promoter	CCCACCGGCCCTTTATAATGCGA	ATACTCAGCGCGATCCCTCCCT	95 °C, 5m
CTCFBS	AGAGCCGAGATTTGAGCCCAGT	GGTCCCTGCCCTTGATTTGCTG	95 °C, 30 s; 65 °C, 30 s; 72 °C, 30s
H19 ICR	ATGAGCGTCCTATTCCCAGA	CTCACACATCACAGCCCAAG	X 36
CCAT1 promoter	CCTCACATGGCTCCCATCACACTAA	CGTGGCAATTACCATGGTCCTTGC	
CTCF negative site	CCCAACATTGCAGCCTCTGA	GGGCTGTCCTCCACCTCTGA	
Mut_seq (D3, E4)	CTAAACCTCTTCATTATTTTATTTCA	TAGTTTAAGGTCAAGCTGTG	95 °C, 30 s; 53 °C, 30 s; 72 °C, 30s x 36
Mut_seq WT	CCTAAACCTCCTCACCATTGGA	AGAGTGAGGGGACATCCTGTAT	95 °C, 30 s; 60 °C, 30 s; 72 °C, 30s x 36
TCF4	TAAATTTGCTGCTGGTGCTG	GGGGTTTTGGAAAGACACAA	*95 °C, 30 s; 65 °C, 30 s; 72 °C, 30 s

PCR primers for DNA FISH and RNA FISH

Primer ID	Forward	Reverse	Cycle
MYC F1, R1	AAGGAACCGCCTGTCCTTCC	CGATCCCTCCCTCCGTTCTT	
MYC F2, R2	CCGGTTTTCGGGGCTTTATC	TCCGGGTCGCAGATGA	94 °C, 2 m
MYC F3, R3	TGTATGTGGAGCGGCTTCTCG	CAGCCAAGGTTGTGAGGTTG	94 °C, 15 s; 60 °C, 15 s; 68 °C, 2-3 m
MYC F4, R4	TGCATGATCAAATGCAACCTCA	CTTCTTCCCAGGAGCCGTCA	x 34
SupE F1, R1	GGCACTTCACACGGATTGCTC	CACTGCACACGGGAAATGCT	
SupE F2, R2	TTTTTCCGGGCTTTGAAAGAT	CTCACCCAAGCTCCCTCAGC	

PCR primers for total RNA and RNA export assay

Primer ID	Forward	Reverse	Cycle
MYC	TACAACACCCGAGCAAGGAC	TTCTCCTCCTCGTCGCAGTA	95 °C, 5 min
MYC intron 1	CGCTGGAAACCTTGCACCTC	CGATCCCTCCCTCCGTTCTT	95 °C, 30 s; 65 °C, 30 s; 72 °C, 30 s
CytB	CCGGTTTTCGGGGCTTTATC	TCCGGGTCGCAGATGA	x 36
TBP	TTCCGCTGGCCCATAGTGAT	TGCTGCTGCCTTTGTTGCTC	
b-Actin	CGTCCCAGTTGGTGACGATG	CCGTGCTCAGGGCTTCTTGT	
ERCC-113	GCGACACCAACATCGTTACG	CCGCGCGTGAGCACTT	
CCAT1	CATTGGGAAAGGTGCCGAG	ACGCTTAGCCATACAGAGCC	
FAM49B	GGGGTGCAGTTGTTCCACTA	CTCGCTCTAGATGCTGGGTG	
CTCF	TTGTGCAGTTATGCCAGCAG	CACTTTGGGTAAACCGAGCA	
AHCTF1	TCAGAAAGGTCCGGCAACAA	CGCCACAGCTTCCTTCACTA	

CRISPR oligonucleotides

guide RNA sequence	UAAACAGCAAUGCCCUCCAA
Donor DNA sequence	TTCTCACTGACTCTAAAACCTATCCATGCTCCTAAACCTCTTCATTATTTTATTTCATTGCTGTTTACCCTTTCAGTTTCAGCTGTACTATCAAAAGCAG
