# Supplementary material for: Canonical WNT signaling-dependent gating of MYC requires a noncanonical CTCF function at a distal binding site
Source: Nat Commun. 2022 Jan 11;13:204. doi: 10.1038/s41467-021-27868-3 (PMC8752836; doi:10.1038/s41467-021-27868-3)
Supplement: Supplementary file 3 — Reporting Summary [file 41467_2021_27868_MOESM3_ESM.pdf]

nature research

Corresponding author(s): Anita Göndör

Last updated by author(s): Nov 12, 2021

Reporting Summary

Nature Research wishes to improve the reproducibility of the work that we publish. This form provides structure for consistency and transparency in reporting. For further information on Nature Research policies, see our Editorial Policies and the Editorial Policy Checklist.

Statistics

For all statistical analyses, confirm that the following items are present in the figure legend, table legend, main text, or Methods section.

n/a	Confirmed	
☐	☒	The exact sample size (*n*) for each experimental group/condition, given as a discrete number and unit of measurement
☐	☒	A statement on whether measurements were taken from distinct samples or whether the same sample was measured repeatedly
☐	☒	The statistical test(s) used AND whether they are one- or two-sided<br>*Only common tests should be described solely by name; describe more complex techniques in the Methods section.*
☒	☐	A description of all covariates tested
☒	☐	A description of any assumptions or corrections, such as tests of normality and adjustment for multiple comparisons
☐	☒	A full description of the statistical parameters including central tendency (e.g. means) or other basic estimates (e.g. regression coefficient) AND variation (e.g. standard deviation) or associated estimates of uncertainty (e.g. confidence intervals)
☐	☒	For null hypothesis testing, the test statistic (e.g. *F*, *t*, *r*) with confidence intervals, effect sizes, degrees of freedom and *P* value noted<br>*Give P values as exact values whenever suitable.*
☒	☐	For Bayesian analysis, information on the choice of priors and Markov chain Monte Carlo settings
☒	☐	For hierarchical and complex designs, identification of the appropriate level for tests and full reporting of outcomes
☒	☐	Estimates of effect sizes (e.g. Cohen's *d*, Pearson's *r*), indicating how they were calculated

Our web collection on statistics for biologists contains articles on many of the points above.

Software and code

Policy information about availability of computer code

Data collection	No software was used for data collection
Data analysis	The pipeline used to map the chromatin networks by Nodewalk analyses is available on request. The simulation of the kinetics of mRNA export used an in-house code, which has been published (ref nr 7 in the manuscript).<br><br>RNA-seq library construction was performed with standard Illumina TruSeq Stranded mRNA kit with Poly-A selection and samples were sequenced on NovaSeq6000 (NovaSeq Control Software 1.7.0/RTA v3.4.4) with a 51nt(Read1)-10nt(Index1)-10nt(Index2)-51nt(Read2) setup using 'NovaSeqStandard' workflow in 'SP' mode flowcell. Data analysis was performed by nf-core RNA-seq pipeline (v.3.0) with default parameter. In brief, adapters and low quality reads were filtered by trimgalore (0.6.6)[https://github.com/FelixKrueger/TrimGalore]. Clean reads were aligned to the GRCh37 human reference genome and ERCC RNA spike-in fasta (https://www.thermofisher.com/se/en/home.html) with hisat2(2.2.0). Genome-wide coverage output in BEDGRAPH format were generated by Bedtools genomecov subcommand (V 2.29.2). Bamcoverage from Deeptools (3.5.1) was to create bigWig coverage files. Integrative Genomics Viewer was used to view the read coverage.<br><br>Genome wide off-target sequencing was analyzed according to a modified method of GOTI. Briefly, low-quality reads and adapter were trimmed by Trimmomatic. BWA was subsequently used to align clean reads to the genome and Picard (https://broadinstitute.github.io/picard/) was used to mark duplicates. To reduce the false positive rate, we applied 3 methods to detect the SNV and Indel between D3/E4 and WT, mutect2, Strelka2 and Lofreq. The overlapping SNVs and Indels were treated as true variants that were further annotated by Annovar. The adjacent 22-bp sequences of the off-target variants were retrieved from the mapping files and blasted with the 22bp sgRNA (19bp sgRNA target sequence and 3bp PAM). High sequence similarities indicated that the off-target variants were sgRNA-associated, while low sequence similarity meant sgRNA-independence. To further screen for potential off-target sites that overlapped with the identified variant we used Cas-OFFinder (http://www.rgenome.net/cas-offinder/).

For manuscripts utilizing custom algorithms or software that are central to the research but not yet described in published literature, software must be made available to editors and reviewers. We strongly encourage code deposition in a community repository (e.g. GitHub). See the Nature Research guidelines for submitting code & software for further information.

Data

Policy information about availability of data

All manuscripts must include a data availability statement. This statement should provide the following information, where applicable:

- Accession codes, unique identifiers, or web links for publicly available datasets
- A list of figures that have associated raw data
- A description of any restrictions on data availability

Data availability

The genomic data reported in this paper has been deposited to general databases with the following accession numbers: For genomic sequences SRA accession nr: PRJNA756713, for ChIP-seq GEO accession nr: GSE184106 and for RNA-seq data GEO accession nr: GSE184103. The source data underlying Figures 1B,D, and E; 2B-I, K; 5B-F; 6C-F, Extended Figures 1A-C; 3A-D and G; 5 and 6 are provided in the Source Data file. All other relevant data supporting the key findings of this study are available within the article and its Supplementary Information files or from the corresponding author upon reasonable request.

Field-specific reporting

Please select the one below that is the best fit for your research. If you are not sure, read the appropriate sections before making your selection.

☒ Life sciences ☐ Behavioural & social sciences ☐ Ecological, evolutionary & environmental sciences

For a reference copy of the document with all sections, see nature.com/documents/nr-reporting-summary-flat.pdf

Life sciences study design

All studies must disclose on these points even when the disclosure is negative.

Sample size	The ChIP-qPCR/ChIP-seq, Nodewalk and RT-qPCR results represent in all cases three independent experiments to enable statistical analyses. Microscopic analyses were performed at least twice and in some instances in three independent experiments. These sample sizes, producing large data sets for the microscopic analyses, are commonly accepted in the community and suitable for statistical analyses comparing distribution of alleles within a population.
Data exclusions	No data was excluded
Replication	All the independent experiments were performed on three or more separate occasions using different cell preparations
Randomization	This is not applicable as the report does not contain any prognostic or predictive information
Blinding	This is not applicable as the report does not contain any prognostic or predictive information

Reporting for specific materials, systems and methods

We require information from authors about some types of materials, experimental systems and methods used in many studies. Here, indicate whether each material, system or method listed is relevant to your study. If you are not sure if a list item applies to your research, read the appropriate section before selecting a response.

Materials & experimental systems

n/a	Involved in the study	
☐	☒	Antibodies
☐	☒	Eukaryotic cell lines
☒	☐	Palaeontology and archaeology
☒	☐	Animals and other organisms
☒	☐	Human research participants
☒	☐	Clinical data
☒	☐	Dual use research of concern

Methods

n/a	Involved in the study	
☐	☒	ChIP-seq
☒	☐	Flow cytometry
☒	☐	MRI-based neuroimaging

Antibodies

Antibodies used

Rabbit anti-NUP133 (Abcam, ab155990)
Mouse anti-TATA binding protein (Abcam, ab51841)
mouseRabbit polyclonal anti-CTCF Cell Signaling Technology 2899S
Mouse monoclonal anti-CTCF BD Biosciences 612148
Mouse monoclonal anti-CTCF Abcam 37477
Rabbit polyclonal anti-AHCTF1 Novusbio NBP1-87952
Rabbit polyclonal anti-AHCTF1 Novusbio NB600-238
Rabbit monoclonal anti-Biotin Cell Signaling Technology 5597S
Mouse monoclonal anti-Digoxigenin Roche 11333062910
Rabbit polyclonal IgG Cell Signaling Technology 2729S
Mouse polyclonal IgG Santa Cruz 2025
Rabbit monoclonal anti-NUP133 Abcam 155990
Rabbit monoclonal anti-β-catenin Cell Signaling Technology 8480S
Rabbit monoclonal anti-TCF4 Cell Signaling Technology 2569S

Validation

All antibodies were approved of only if they detected the correct bands upon WES/JESS analyses

Eukaryotic cell lines

Policy information about cell lines

Cell line source(s)	The primary cultures of human colon epithelial cells were purchased from ScienCell. The HCT-116 cell line was obtained from professor B Vogelstein and are available commercially at ATCCC (91091005-1VL).
Authentication	The HCT-116 cells used in this study has undergone STR authentication by ATCC
Mycoplasma contamination	The cells were routinely tested for mycoplasma contamination. In no instance have the cells used in this manuscript been compromised by any such infection.
Commonly misidentified lines (See ICLAC register)	No commonly misidentified cell line was used

ChIP-seq

Data deposition

☒ Confirm that both raw and final processed data have been deposited in a public database such as GEO.

☒ Confirm that you have deposited or provided access to graph files (e.g. BED files) for the called peaks.

Data access links
May remain private before publication.

For genomic sequences SRA accession nr: PRJNA756713, for ChIP-seq GEO accession nr: GSE184106 and for RNA-seq data GEO accession nr: GSE184103

Files in database submission

For genomics sequences SRA data accession:

WT: SRR15559958
E4: SRR15559959
D3: SRR15559960

For RNA-seq data GEO accession:
GSM5577953_D3_1.coverage.bw
GSM5577954_E4_1.coverage.bw
GSM5577955_WT_1.coverage.bw
GSM5577956_D3_2.coverage.bw
GSM5577957_E4_2.coverage.bw

For ChIP-seq data GEO accession:

GSM5577970_WT_CTCF_R1_peaks.broadPeak.gz
GSM5577971_WT_CTCF_R2_peaks.broadPeak.gz
GSM5577972_WT_CTCF_R3_peaks.broadPeak.gz
GSM5577973_D3_CTCF_R1_peaks.broadPeak.gz
GSM5577974_D3_CTCF_R2_peaks.broadPeak.gz
GSM5577975_D3_CTCF_R3_peaks.broadPeak.gz
GSM5577976_E4_CTCF_R1_peaks.broadPeak.gz
GSM5577977_E4_CTCF_R2_peaks.broadPeak.gz

GSM5577978_E4_CTCF_R3_peaks.broadPeak.gz

Genome browser session (e.g. UCSC)

http://genome-euro.ucsc.edu/s/wushyer/HCT116-ChipSeq-II

Methodology

Replicates

All ChIP-seq datatsets are derived from 3 biological replicates

Sequencing depth

All the sample is paired-end library with length 2X150 bp with ranging from 19-33Mb reads. Detailed statistics listed below

Library Total_ reads Aligned_reads Unique_mapQ>30 Run Depth
D3_CTCF_R1 25826058 18593110 18449393 paired-end-150bp 1.251386191
D3_CTCF_R2 20881960 14970030 14845921 paired-end-150bp 1.01182288
D3_CTCF_R3 22012936 16046778 15916588 paired-end-150bp 1.066623646
D3_no_ab_R1 29906358 11435552 11251975 paired-end-150bp 1.449094687
D3_no_ab_R2 19537306 10079346 9858098 paired-end-150bp 0.946668475
D3_no_ab_R3 24178546 13506178 12939964 paired-end-150bp 1.171556983
E4_CTCF_R1 28904450 20556830 20388988 paired-end-150bp 1.400547834
E4_CTCF_R2 22062814 15879156 15750834 paired-end-150bp 1.069040454
E4_CTCF_R3 19796104 15125694 15009827 paired-end-150bp 0.959208377
E4_no_ab_R1 29791320 6964290 6811740 paired-end-150bp 1.443520589
E4_no_ab_R2 25391456 16682004 16512739 paired-end-150bp 1.230327811
E4_no_ab_R3 27931148 14930008 14724067 paired-end-150bp 1.353387068
WT_CTCF_R1 22838594 16532104 16398170 paired-end-150bp 1.10663041
WT_CTCF_R2 24604534 17520258 17378043 paired-end-150bp 1.192197976
WT_CTCF_R3 25515504 18779954 18633393 paired-end-150bp 1.236338483
WT_no_ab_R1 33021860 18003780 17826949 paired-end-150bp 1.600054473
WT_no_ab_R2 22164796 12808532 12654057 paired-end-150bp 1.073981931
WT_no_ab_R3 20595282 10779816 10547569 paired-end-150bp 0.997932069

Antibodies

CTCF (Cell Signaling Technology; CS 2899S)

Peak calling parameters

BWA was used for reads mapping and Macs2 was used for peak calling. All the command line was implemented in nf-core/chip-seq.1.2.2 with CTCF treated as IP and no antibody as input. The command line is “nextflow run ../software/chipseq-1.2.2/ -profile singularity -with-singularity ../software/nfcore-chipseq.simg --genome GRCh37 --input read.csv --outdir ./0719”

Data quality

MACS2 FRiP score is generated by calculating the fraction of all mapped reads that fall into the MACS2 called peak regions. Phantompeakqualtools was further used to estimate the relatively strand cross-correlation coefficient in all samples.
Library FRiP score Total_peaks FDR 5% and >5-fold enrichment
D3_CTCF_R1 0.42939 57935 24535
D3_CTCF_R2 0.41517 57475 24495
D3_CTCF_R3 0.393704 51715 23303
E4_CTCF_R1 0.279027 39434 19611
E4_CTCF_R2 0.419365 51422 20838
E4_CTCF_R3 0.434209 48235 19682
WT_CTCF_R1 0.156481 38645 19045
WT_CTCF_R2 0.203918 41380 20779
WT_CTCF_R3 0.170255 38655 17544

Relatively strand cross-correlation coefficient in each library

D3_CTCF_R1 1.106373
D3_CTCF_R2 1.202278
D3_CTCF_R3 1.230677
D3_no_ab_R1 0.6345716
D3_no_ab_R2 0.4524308
D3_no_ab_R3 0.2726278
E4_CTCF_R1 1.245693
E4_CTCF_R2 1.231754
E4_CTCF_R3 1.210388
E4_no_ab_R1 0.5749267
E4_no_ab_R2 0.967061
E4_no_ab_R3 0.623728
WT_CTCF_R1 1.254182
WT_CTCF_R2 1.406901
WT_CTCF_R3 1.612281
WT_no_ab_R1 1.514352
WT_no_ab_R2 1.052192
WT_no_ab_R3 0.4194285

Software

Chip-seq data analyses were performed by nf-core/chipseq pipeline (version 1.2.2;). Briefly, Trim Galore (https://www.bioinformatics.babraham.ac.uk/projects/trim_galore/) was used to trim adapter. BWA was used to perform read alignment. Picard (http://broadinstitute.github.io/picard) was used to mark duplicates. BEDTools and bedGraphToBigWig were used create normalized bigWig files. Phantompeakqualtools was used to calculate strand cross-correlation peak and ChIP-seq quality measures. MACS2 was used to call peaks and HOMER was used to perform peak annotation. All the read coverages were visualized by IGV genome browser version 2.5.3. For quantitation of microscopic images Imaris v.8.1.2 was used.
